# Predictive models for mutations in mismatch repair genes: implication for genetic counseling in developing countries

**DOI:** 10.1186/1471-2407-12-64

**Published:** 2012-02-09

**Authors:** Erika Maria Monteiro Santos, Mev Dominguez Valentin, Felipe Carneiro, Ligia Petrolini de Oliveira, Fabio de Oliveira Ferreira, Samuel Aguiar Junior, Wilson Toshihiko Nakagawa, Israel Gomy, Victor Evangelista de Faria Ferraz, Wilson Araujo da Silva Junior, Dirce Maria Carraro, Benedito Mauro Rossi

**Affiliations:** 1 Graduation Program, AC Camargo Hospital, Sao Paulo, Brazil; 2 International Center of Research and Training (CIPE), AC Camargo Hospital, Sao Paulo, Brazil; 3 Hereditary Colorectal Cancer Registry, AC Camargo Hospital, Sao Paulo, Brazil; 4 Sao Paulo University, Department of Genetics, Medical School of Ribeirao Preto, Ribeirao Preto, Brazil

## Abstract

**Background:**

Lynch syndrome (LS) is the most common form of inherited predisposition to colorectal cancer (CRC), accounting for 2-5% of all CRC. LS is an autosomal dominant disease characterized by mutations in the mismatch repair genes mutL homolog 1 (MLH1), mutS homolog 2 (MSH2), postmeiotic segregation increased 1 (PMS1), post-meiotic segregation increased 2 (PMS2) and mutS homolog 6 (MSH6). Mutation risk prediction models can be incorporated into clinical practice, facilitating the decision-making process and identifying individuals for molecular investigation. This is extremely important in countries with limited economic resources. This study aims to evaluate sensitivity and specificity of five predictive models for germline mutations in repair genes in a sample of individuals with suspected Lynch syndrome.

**Methods:**

Blood samples from 88 patients were analyzed through sequencing MLH1, MSH2 and MSH6 genes. The probability of detecting a mutation was calculated using the PREMM, Barnetson, MMRpro, Wijnen and Myriad models. To evaluate the sensitivity and specificity of the models, receiver operating characteristic curves were constructed.

**Results:**

Of the 88 patients included in this analysis, 31 mutations were identified: 16 were found in the MSH2 gene, 15 in the MLH1 gene and no pathogenic mutations were identified in the MSH6 gene. It was observed that the AUC for the PREMM (0.846), Barnetson (0.850), MMRpro (0.821) and Wijnen (0.807) models did not present significant statistical difference. The Myriad model presented lower AUC (0.704) than the four other models evaluated. Considering thresholds of ≥ 5%, the models sensitivity varied between 1 (Myriad) and 0.87 (Wijnen) and specificity ranged from 0 (Myriad) to 0.38 (Barnetson).

**Conclusions:**

The Barnetson, PREMM, MMRpro and Wijnen models present similar AUC. The AUC of the Myriad model is statistically inferior to the four other models.

## Background

Lynch syndrome (LS) is the most common form of inherited predisposition to colorectal cancer (CRC), accounting for 2-5% of all CRC [1]. Colorectal cancer in LS differs from sporadic cases by an earlier age of diagnosis (mean age approximately 44 years), a predominance of proximally-sited colon cancers (60-70%) and an increased propensity to synchronous or metachronous CRCs (25%) [2,3]. Individuals with LS have an 80% probability of developing CRC at 65 years, and they are at an elevated risk of developing a second primary CRC [4] as well as at an increased risk for extra-colonic malignancies, including endometrial, gastric, small bowel, urological tract, ovary, pancreas and brain cancer [5].

Family history has been the primary method for identifying patients at risk. The Amsterdam criteria, the first formal guidelines for the clinical diagnosis of LS, is based on the presence of: (a) three or more relatives with CRC, one of whom is the first-degree relative of the other two; (b) cancer in at least two generations of the same family; and (c) at least one cancer case diagnosed before the age of 50 [6]. These criteria, later, evolved to become the Amsterdam II criteria, which included extracolonic malignancies, such as endometrial, small bowel, renal pelvic and ureter cancers [7]. The Amsterdam criteria I/II, although specific, showed little sensitivity (probability that a test result will be positive when a disease is present [8]), which led to the establishment of the Bethesda guidelines, which helped the identification of individuals who should be considered at risk and need further evaluation of microsatellite instability (MSI) and genetic testing [9,10]. The Bethesda criteria showed to be more sensitive, but its specificity (probability that a test result will be negative when the disease is not present [8]) decreased substantially.

LS is an autosomal dominant disease characterized by mutations in the mismatch repair genes mutL homolog 1 (MLH1), mutS homolog 2 (MSH2), postmeiotic segregation increased 1 (PMS1), post-meiotic segregation increased 2 (PMS2) and mutS homolog 6 (MSH6) [7-11]. Germline abnormalities in MLH1 and MSH2 genes are found in more than 90% of LS mutation carriers [12].

The majority of MMR (mismatch repair) gene mutations in LS patients cause truncations and loss of function of the affected polypeptide [13-15]. To date, over 513 different DNA alterations have been reported, the majority of which are represented by single nucleotide substitutions, deletions or insertions [16]. However, amino acid alterations comprise a significant proportion of the mutations (~10% of *MSH2 *and ~30% of *MLH1*). These types of alterations are better known as variants of uncertain significance (VUS), often called unclassified variants (UVs) [13-15]. Our ability to better select a non-synonymous single-nucleotide polymorphism (nsSNP) for an association study can be enhanced by first examining the potential impact an amino acid variant may have on the function of the encoded protein with the use of two innovative sequence homology-based programs, Sort Intolerant from Tolerant (SIFT) and Polymorphism Phenotype (PolyPhen-2). SIFT uses sequence homology among related genes and domains across species to predict the impact of all 20 possible amino acids at a given position, allowing users to determine which nsSNPs would be the most interesting to study. PolyPhen also takes an evolutionary approach, but differs from SIFT in that it predicts how damaging a particular variant may be by using a set of empirical rules based on sequence, phylogenetic, and structural information [17].

Regarding social aspects, both economic and ethical, pre-test genetic counselling performed by specialized professionals is crucial for the discussion of benefits and limitations of genetic testing [18], particularly in countries with limited resources. Since the estimated probability of mutation is an important component of genetic counselling, models predicting the risk of mutation can be incorporated into clinical practice, facilitating the decision-making process, and help to better identify individuals for molecular investigation [19]. In addition, prediction models can be used to identify individuals at high risk for cancer, who might benefit from interventions, as well as to develop risk-benefit indexes and to estimate the impact of the disease [19]. After the first model published to evaluate the risk of chronic disease in 1976, numerous models related to the risk of developing cancer have been created, and with the discovery of the genetic susceptibility to breast cancer, models to assess the likelihood of an individual carrying a germline mutation have been widely used.

According to Freedman et al. [19] models should have high sensitivity since it is assumed that they should identify all mutation carriers. However, high specificity is also important, especially from the clinical and economical point of view, since it does not want to subject individuals with a low likelihood to develop the disease to the risks and costs of genetic testing.

Up until now, models predicting the risk of germline mutations in patients with CRC were evaluated in consecutive samples [20-27] and in patients at high risk due to the positive family history [28-30]. In these studies, samples from Latin American populations were always underrepresented. Therefore, it is important to assess the accuracy of the models currently available in Latin America populations, since they may have direct implications in the clinical practice and genetic counselling.

This study aims to evaluate the sensitivity (probability that a test result will be positive when the disease is present) and specificity (probability that a test result will be negative when the disease is not present) of five predictive models for germline mutations in mismatch repair genes in a sample of individuals with suspected Lynch syndrome.

## Methods

### Patient selection

The patients were recruited from two institutions in Sao Paulo state, southeastern Brazil, who had participated in a project that evaluated the Lynch syndrome profile in South America. Eighty-eight individuals, whose clinical information and heredogram were addressed, with colorectal cancer that fulfilled the Bethesda guidelines, were included. This project was approved by the Institutional Review Board at A.C. Camargo Hospital. After genetic counseling and signature of informed consent, peripheral blood samples of subjects were collected.

The patients' demographic, clinical and mutation characteristics are shown in Table 1. Ethnicity was classified according to the patient's self-report, according to the recommendations of the IBGE (Brazilian Institute of Geography and Statistics) [31], most patients self-declared white (62.5%). Most patients were born in the Southeast (76%). The average age at diagnosis of colorectal cancer was 42.37 years, and the mean age at diagnosis of endometrial cancer was 46.17 years. Of the total, 21 individuals presented more that two primary tumors.

**Table 1:** Demographic, clinical and mutation characteristics of the sample

Characteristic	Category	N	(%)
Age at CRC diagnosis	Under 30 yrs old	13	14.8
	31-50 yrs old	54	61.4
	Over 50 yrs old	21	23.9
Sex	Female	58	65.9
	Male	30	34.1
Ethnicity (self-reported)	White	55	62.5
	"Pardo"*	31	35.2
	Non-available	2	2.3
Place of Birth	Southeast	67	76.1
	Northeast	12	13.6
	South	3	3.4
	North	2	2.3
	Midwest	2	2,3
Colorectal tumor	Separate	78	88.6
	Synchronous	9	10.2
	Metachronous	1	1.1
Extracolonic tumors in proband	Endometrial	6	6.8
	Breast	3	3.4
	Stomach	2	2.3
	Small intestine	2	2.3
	Hepatic	1	1.1
	Pelvis renal and ureter	1	1.1
	Ovary	1	1.1
Classification according to family history	Bethesda Criteria	50	56.8
	Amsterdam Criteria	38	43.2
Patogenic mutations	None	57	64.8
	MLH1	15	17.0
	MSH2	16	18.2

*Those who declare with admixture or multiethnic origin

### Sequencing

Genomic DNA sequences of the MLH1, MSH2 and MSH6 genes were obtained from the NCBI Nucleotide database (NM_000249.2 NM_000251.1 and NM_000179, respectively). Sequencing was performed in a 3130xl Genetic Analyzer (Applied Biosystems).

The mutations found were confirmed in a second sample through another PCR reaction with the use of Platinum^® ^Taq DNA Polymerase High Fidelity (Invitrogen, São Paulo, Brazil), followed by sequencing.

For the interpretation and determination of the pathogenicity of mutations, the following databases were consulted: INSIGHT (International Society for Gastrointestinal Hereditary Tumors); MMR Gene Unclassified Variants Database; Mismatch Repair Genes Variant Database and the Human Gene Database at the Institute of Medical Genetics in Cardiff.

For the non-described variants: nonsense mutations, small duplications, deletions and insertions have been classified as pathogenic mutations, since they cause the interruption of the protein reading frames; those mutations that affect donor splicesites have also been classified as pathogenic since they affect the mRNA splicing process, consequently affecting the protein structure.

For the pathogenicity prediction of missense variants, two complementary algorithms were used: PolyPhen 2(Polymorphism Phenotyping) and SIFT (Sorting Intolerant from Tolerant). SIFT values range from 0 to 1: scores ≤ 0.05 were considered intolerant or substitution of deleterious amino acid, while scores above 0.05 were considered as tolerant. Structural changes levels were determined by applying PolyPhen-2. PolyPhen-2 values range from 0 to 3.37: variants with scores ≥ 2 were considered as "probably damaging," scores between 1.50 and 1.99 indicated a "possibly damaging," variant and scores 0 to 1.49 were considered as benign variants.

From 88 unrelated patients, 31 patogenic mutations were identified, 11 for the first time [5]. The most frequent mutation was c.2152 C > T; p.Gln718X in MSH2 that was observed in six subjects. All the other mutations were observed once. From 15 MLH1 mutations, 3 were missense, 6 nonsense, 3 splice site and 3 frameshift. From 16 MSH2 mutations, one was missense, 10 nonsense, 1 splice site, and 4 frameshift.

### Calculation of risk models

We consulted clinical information from patients' records and pedigrees to fit the risk prediction models. The probability of identifying a mutation was calculated by five models: PREMM (Prediction of Mutations in MLH1 and MSH2) [23]; Barnetson [21]; MMRpro [20]; Wijnen [22]; and Myriad [24] were calculated using the CancerGene software program (The University of Texas Southwestern Medical Center, Dallas, USA).

The PREMM model was developed from a multiple logistic regression model in a cohort of 898 individuals, and subsequently was validated in 1016 patients. This model accounts for: the presence of CRC in the proband, age at diagnosis and the presence of multiple tumors; the occurrence of endometrial tumors and other Lynch syndrome-associated tumors in the proband; occurrence of CRC in first-and second-degree relatives, the number of affected relatives and the age at diagnosis of the youngest individual; the presence of endometrial tumors and Lynch syndrome-associated tumors in first-and second-degree relatives [20].

The Barnetson model is based on the analysis of the characteristics of 870 patients with less than 55 years of age with CRC. This model enables the inclusion of microsatellite instability and immunohistochemistry (IHQ). The other variables included in this model are: age at diagnosis of CRC; tumor location; occurrence of synchronous and metachronous tumors; first-degree relatives with CRC and endometrial cancer [21].

MMRpro uses a Bayesian method for calculating the probability of mutation and also provides the risk of CRC and endometrial cancer for non-affected individuals. Information used for the calculation of probability includes: age of affected and non-affected relatives; age at diagnosis of CRC; age at diagnose of endometrial cancer; result of microsatellite instability or IHQ if the tumor is available; molecular diagnostic result if performed [20].

The Wijnen or Leiden model was developed from the analysis of 184 families with colorectal cancer. Variables of this model are: age at diagnosis of CRC; presence of endometrial tumor in family; fulfilment of Amsterdam criteria [22].

Myriad elaborated mutation prevalence tables from the analysis of 3410 individuals. The occurrence of CRC, endometrial cancer and other tumors related to Lynch syndrome are considered [24].

### Statistical analysis

To evaluate the sensibility and specificity of the models, receiver operating characteristic (ROC) curves were constructed by choosing cutpoints and computing the sensitivity against specificity. The area under the ROC curve describes the model discriminatory ability of distinguishing carriers from non-carriers. An AUC (area under the curve) of 0.5 indicates the absence of discriminatory ability, and 1 indicates a perfect discrimination [32,33]. To compare the models, the method described by DeLong et al. was used [34]: where areas under correlated ROC curves are compared through a non-parametric approach that applied generalized U-statistics on the covariance matrix estimation.

Taking into consideration that genetic counselling guidelines recommended genetic testing for breast cancer patients if a risk prediction model estimates a probability to detect a mutation ranges from 10% to 20% [35], we calculate sensitivity and specificity for all models at 5%, 10%, 20% and 30%.

We also evaluated personal and family history characteristics in our data that could predict mutation status. Chi-square test was used for categorical data and student-*t *test for numeric data in order to identify changes in means of the subpopulations. Variables with a *p*-value < 0.20 were selected for multiple analyses. Logistic Regression Analysis was performed with calculation of Odds Ratio (OR), considering the identification of pathogenic mutation as the outcome. The *p*-value of 0.05 was considered statistically significant. All statistical analysis was performed using MedCalc version 11 and SPSS for Windows version 15.

## Results

Of the 38 patients who fulfilled the Amsterdam criteria, 23 presented mutations; 8 patients with Bethesda Guidelines presented mutations in the MLH1 or MSH2 genes. The sensitivity of the Amsterdam criteria was 0.74 and the specificity was 0.73 the sensitivity and specificity of Bethesda Criteria were not calculated since it was the inclusion criteria for this study.

**Figure 1:**
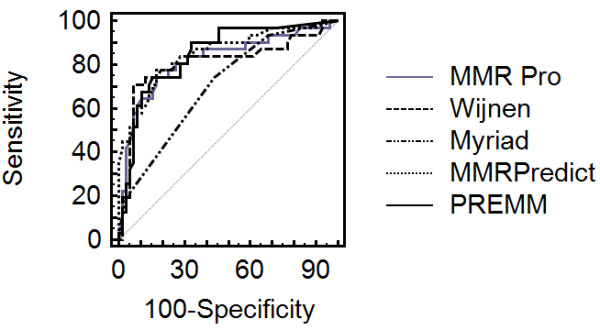
**ROC Curve of risk prediction models for MMR germline mutations**.

Figure 1 presents the ROC curve. Table 2 presents the AUC of models for pathogenic mutation and Table 3 the Pairwise comparison of ROC curves for MMR germiline mutation. The AUC for the PREMM, Barnetson, MMRpro and Wijnen models did not present significant statistical difference. The Myriad model presented a lower AUC than the four other models evaluated (*p *< 0.005) according to the method to compare ROC curves described into Methods section.

**Table 2:** AUC of risk prediction models, standard error (SE) and 95% CI

Model	AUC	SE	95%CI	Sensitivity	Specificity
PREMM	0.846	0.0476	0.753-0.914	0.74	0.82
MMRPredict	0.850	0.0471	0.758-0.917	0.77	0.82
MMRpro	0.821	0.0506	0.725-0.895	0.74	0.82
Wijnen	0.807	0.0567	0.709-0.883	0.71	0.93
Myriad	0.704	0.0604	0.598-0.797	0.74	0.56

**Table 3:** Pairwise comparison of ROC curves for MMR germiline mutation

	Models									
Models	MMRPro		MMRPredict		Myriad		PREMM		Wijnen	
	Dif*	p	Dif*	p	Dif*	p	Dif*	p	Dif*	p
MMRPRo			0.028	0.524	0.117	0.017	0.024	0.479	0.014	0.762
MMRPredict	0.028	0.524			0.145	0.039	0.003	0.885	0.042	0.401
Myriad	0.117	0.017	0.145	0.003			0.141	0.005	0.103	0.134
PREMM	0.024	0.479	0.003	0.885	0.141	0.005			0.038	0.356
Wijnen	0.014	0.769	0.042	0.401	0.103	0.134	0.038	0.356		

*Dif Difference between areas

Table 4 presents the sensitivity and specificity within 5%, 10%, 20% and 30% thresholds of the risk prediction models for the MMR germiline mutations. Considering the ≥ 10% the sensitivity among models ranges from 0.90 (MMRPredict) to 0.96 (MMRPro, Myriad, Wijnen) and the specificity ranges from 0.08 (MMRPro) to 0.54 (PREMM).

**Table 4:** Sensitivity and specificity within the ≥ 5%, ≥ 10%, ≥ 20% and ≥ 30% thresholds of the risk prediction models for the MMR germline mutation

Models and threshold	Sensitivity	Specificity
MMRpro		
≥ 5%	1.00	0.03
≥ 10%	0.96	0.08
≥ 20%	0.90	0.38
≥ 30%	0.87	0.52
MMRPredict		
≥ 5%	0.93	0.38
≥ 10%	0.90	0.33
≥ 20%	0.83	0.70
≥ 30%	0.80	0.75
Myriad		
≥ 5%	1.00	0
≥ 10%	0.94	0.31
≥ 20%	0.74	0.56
≥ 30%	0.10	0.92
PREMM		
≥ 5%	0.98	0.28
≥ 10%	0.90	0.54
≥ 20%	0.67	0.85
≥ 30%	0.67	0.85
Wijnen		
≥ 5%	1.00	0
≥ 10%	0.94	0.31
≥ 20%	0.74	0.56
≥ 30%	0.10	0.92

Table 5 presents results from AUC for risk prediction models for MLH1 mutation and Table 6 the Pairwise comparison for MLH1 mutation. Table 7 and 8 presents, respectively, regarding AUC and Pairwise comparison for MSH2 mutation.

**Table 5:** AUC of risk prediction models for MLH1 mutation, standard error (SE) and 95% CI

Model	AUC	SE	95%CI	Sensitivity	Specificity
PREMM	0.721	0.0587	0.599-0.843	1	36.1
Barnetson	0.796	0.0562	0.696-0.874	0.87	0.61
MMRpro	0.742	0.0642	0.641-0.832	0.81	0.58
Wijnen	0.688	0.0892	0.533-0.741	0.56	0.76
Myriad	0.688	0.0632	0.580-0.782	0.81	0.51

**Table 6:** Pairwise comparison of ROC curves for MLH1 germiline mutation

	Models									
Models	MMRPro		MMRPredict		Myriad		PREMM		Wijnen	
	Dif*	p	Dif*	p	Dif*	p	Dif*	p	Dif*	p
MMRPRo			0.050	0.423	0.056	0.402	0.009	0.844	0.103	0.119
MMRPredict	0.050	0.423			0.108	0.104	0.003	0.885	0.042	0.401
Myriad	0.056	0.402	0.108	0.104			0.066	0.318	0.154	0.014
PREMM	0.009	0.844	0.003	0.885	0.066	0.318			0.112	0.052
Wijnen	0.103	0.119	0.042	0.401	0.154	0.014	0.112	0.052		

*Dif Difference between areas

**Table 7:** AUC of risk prediction models for MSH2 mutation, standard error (SE) and 95% CI

Model	AUC	SE	95%CI	Sensitivity	Specificity
PREMM	0.794	0.0646	0.691-0.891	0.80	0.79
Barnetson	0.793	0.0764	0.650-0.839	0.80	0.69
MMRpro	0.794	0.0794	0.596-0.893	0.73	0.76
Wijnen	0.846	0.0499	0.754-0.914	0.93	0.78
Myriad	0.632	0.0733	0.522-0.732	0.93	0.26

**Table 8:** Pairwise comparison of ROC curves for MSH2 germiline mutation

	Models									
Models	MMRPro		MMRPredict		Myriad		PREMM		Wijnen	
	Dif*	p	Dif*	p	Dif*	p	Dif*	p	Dif*	p
MMRPRo			0.028	0.524	0.129	0.049	0.029	0.515	0.084	0.166
MMRPredict	0.028	0.524					0.037	0.363	0.092	0.120
Myriad	0.129	0.049	0.121	0.091			0.159	0.015	0.214	0.005
PREMM	0.029	0.515	0.037	0.363	0.159	0.015			0.055	0.158
Wijnen	0.084	0.166	0.092	0.120	0.214	0.005	0.055	0.158		

*Dif Difference between areas

Table 9 presents the Logistic Regression Model for identification of pathogenic mutation, considering those personal and family history characteristics that were significant at univariate analysis (data not shown). The model presents a determination coefficient of 0.450 and was statistically significant.

**Table 9:** Logistic regression model from personal and cancer family history characteristics associated with MLH1 or MSH2 pathogenic mutation

Variable	Category	OR	95%CI	*p*-value
CRC location (proband)	Distal	1		
	Proximal	3.616	1.185-11.037	0.024
CRC Histological Type	Tubular Adenocarcinoma	1		
	Mucinous Adenocarcinoma	3.974	1.160-13.613	0.028
Number of CRCs*		1.544	1.172-2.033	0.002

*Based on family history, considered as discrete variable

## Discussion

The identification of MMR mutation carriers is relevant, as there are individuals at high risk of developing cancer and can benefit from follow-up recommendations for the early detection of cancer. However, the cost of mutation detection by DNA sequencing is high, which creates the need for adopting strategies in order to reduce cost but maintain effectiveness. Although microsatellite instability (MSI) and immunohistochemistry have been incorporated into clinical practice (despite the fact that MSI is not a test widely available in Brazil) risk prediction models can be valuable tools that could be integrated in clinical practice.

According to the literature, this study presents a significant sample of individuals from South America [1,5,36-41]. The number of mutations identified in the MSH2 gene was similar to the number of MLH1 mutations found. It was also observed that the most frequent extracolonic tumor in probands was endometrial tumor followed by breast cancer.

Weitzel et al. [42] have pointed out risk prediction model applications that could be perfectly adapted in this situation: risk prediction models could help in the elaboration of reports to health insurance companies in order to get approvals for genetic testing; to provide realistic expectations to the patient regarding a positive result and to reinforce the absence of indication for genetic testing when a low probability mutation probability is calculated together with other screening techniques, such as microsatellite instability. However, for their use in the clinical practice, it is necessary that accuracy and predictive ability is thoroughly evaluated, since they may influence not only the adoption of a particular model but also its threshold.

In choosing the model for use in clinical practice three main points should be considered: the availability of the model; practical aspects in the management of the data; and the model performance.

All models are available via the Internet or through free software. When considering the advantages against time, the PREMM and Barnetson models are the best choice since they demand less time to be filled out. Conversely, MMRpro and Wijnen are available at the genetic counselling package CancerGene (CaGene) and a family's pedigree must be built, which is time consuming. In addition, the information available on CaGene must be stored and retrieved if novel elements from family history appear, demanding recalculations. As such, the structure and availability of human resources also influence the choice of the model.

Regarding the analysis of accuracy, all models used in this series presented AUC superior to 0.5. The largest AUC was from the Barnetson model, but this difference was not significant when compared to PREMM, MMRpro and Wijnen. Both Barnetson and MMRpro use information based on microsatellite instability and IHQ data, which can increase accuracy. In this study, since tumor samples of all subjects had not been taken, it was opted not to include this information. The Myriad model presented an AUC inferior to the four other models, a result also noticed by Monzon et al [28].

Since there is no consensus in the literature about which threshold should be used in these models in order to better indicate molecular investigation, sensitivity and specificity of the five models were calculated according to ≥ 5%, ≥ 10%, ≥ 20% and ≥ 30% threshold. What is often observed is a variation in sensitivity and specificity according to the threshold and model used, which suggests that the use of a single threshold for all models (e.g., 10%) implies the alteration of both sensitivity and specificity which could lead to a different detection mutation rate. With the same threshold and similar sensitivities, as it occurs with a 10% threshold and 0.90 sensitivity by the Barnetson and PREMM models, the specificities are 0.33 and 0.58 respectively. When we consider threshold of 10%, the best relationship between sensitivity and specificity among models is regarding the PREMM model (sensitivity 90% specificity 54%).

As a consequence the healthcare professional must consider the characteristics and the performance of each model. The characteristics of each model should also be discussed and considered prior implementation in a genetic counselling practice.

The cost-effective analysis regarding Lynch Syndrome in South America is a point of debate. These studies are still at the initial stages. The evaluation of the costs will be of crucial importance for the implementation of public policies for genetic testing and management of risk individuals.

Considering the characteristics associated with a pathogenic mutation in our sample, the histologic type was the only that were not included in the models evaluated. It should be considered that this characteristic is not always available at the time for genetic counselling, therefore its inclusion could generate incomplete data.

The Brazilian population is extremely heterogeneous, the result of five centuries of the integration of individuals from three continents: Europeans, Africans and Amerindians [43,44]. After colonization by the Portuguese, Brazil received a significant number of Africans. With the end of slavery, Brazil received European immigrant groups, which contributed to a strong European influence in the genome of the Brazilian citizen. According to Pena and colleagues [43], genetic variation among Brazilians is so broad, that it should not be considered as a group but at the individual level.

Our study has limitations that should be considered. The molecular investigation was limited to direct sequencing, and other techniques were not used for molecular evaluation, such as MLPA, which could have increased the number of identified mutations.

## Conclusion

The Barnetson, PREMM, MMRpro and Wijnen models present similar AUC. The AUC of the Myriad model is statistically inferior to the four other models. When considering a threshold of ≥ 5%, the models sensitivity varied between 1 (Myriad) and 0.87 (Wijnen) and the specificity ranged from 0 (Myriad) to 0.38 (Barnetson). With the threshold of ≥ 10%, the models sensitivity ranged from 0.83 (Wijnen) to 0.96 (MMRpro) and specificity from 0.08 (MMRpro) to 0.54 (PREMM).

## Competing interests

The authors declare that they have no competing interests.

## Authors' contributions

EMMS conceived of the study, and participated in its design, recruited patients, collected clinical data, performed the statistical analysis, and draft the manuscript. MDV carried out the molecular genetic studies, assisted in the writing of the manuscript. FC carried out the molecular genetic studies, assisted in the writing of the manuscript. FOF participated in study design, recruited patients, assisted in the writing of the manuscript. SAJ participated in study design, recruited patients, assisted in the writing of the manuscript. WTN participated in study design, recruited patients, assisted in the writing of the manuscript. IG participated in study design, assisted in the writing of the manuscript. VEFF participated in study design, assisted in the writing of the manuscript. WASJ participated in study design, assisted in the writing of the manuscript. DMC participated in study design, assisted in the writing of the manuscript. BMR conceived of the study, and participated in its design, recruited patients and draft the manuscript. All authors read and approved the final manuscript.

## Pre-publication history

The pre-publication history for this paper can be accessed here:

http://www.biomedcentral.com/1471-2407/12/64/prepub
